# Identification of Functional Variants Between Tong Sheep and Hu Sheep by Whole-Genome Sequencing Pools of Individuals

**DOI:** 10.3390/ijms252312919

**Published:** 2024-11-30

**Authors:** Xiaoqin Tang, Shuhui Wang, Xiaohua Yi, Qi Li, Xiuzhu Sun

**Affiliations:** 1 College of Animal Science and Technology, Northwest A&F University, Yangling, Xianyang 712100, China; txq@nwafu.edu.cn (X.T.); yixiaohua@nwafu.edu.cn (X.Y.); awdhey@gmail.com (Q.L.); 2 College of Grassland Agriculture, Northwest A&F University, Yangling, Xianyang 712100, China

**Keywords:** Pool-seq, Tong sheep, Hu sheep, MAS, miRNA

## Abstract

Tong sheep, known for their superior meat quality and disease resilience, face breeding challenges due to low prolificacy, unlike Hu sheep, which exhibit higher fertility and growth rates. This study identified over 700,000 genetic variants between these breeds through pooled whole-genome sequencing. Functional analysis reveals key differences in pathways related to fat metabolism, insulin signaling, and cell cycle regulation. Notable findings include unique microRNA variants (miR-1185-3p in Tong sheep and miR-487-5p in Hu sheep), with the miR-487-5p mutation potentially regulating KITLG, a fertility-related gene. These results suggest that non-coding RNA mutations contribute to phenotypic differences and provide a genomic foundation for molecular-assisted selection to improve Tong sheep breeding programs.

## 1. Introduction

Domestic sheep have a long history of domestication, spanning over 10,000 years, and have diversified into numerous breeds worldwide [1]. Among these, Tong sheep, a semi-fine wool breed indigenous to China, are highly valued for their wool, minimal mutton odor, distinctive fat tail, and robust immunity. Despite these advantages, Tong sheep’s low reproductive rate, particularly their reduced twin birth rate and mating challenges due to their large fat tails, limits their utility in modern agriculture. Conversely, Hu sheep, another esteemed Chinese breed, demonstrate superior fertility and growth rates [2,3]. This study hypothesizes that distinct genomic variations underlie the phenotypic differences between Tong sheep (T) and Hu sheep (H), particularly in traits related to fertility and fat deposition. Unraveling the genomic discrepancies between breeds with extreme phenotypic traits could enhance the conservation and sustainable use of Tong sheep. Pool-seq reduces the number of redundant reads, making it a more economical approach for genome-wide population genetic studies. Despite the need to account for sequencing errors, pooling often outperforms individual sequencing in estimating allele frequencies and inferring population genetic parameters. Pooling often provides more accurate allele frequency estimates and genetic parameter inference than individual sequencing, despite the need to account for sequencing errors. This study employs the cost-effective benefits of next-generation sequencing (NGS) while minimizing financial constraints, making it an efficient approach for large-scale genetic analysis. The effectiveness of pooling methods has been validated by Van Tassell et al., who demonstrated strong correlations between allele frequency estimates derived from pooled NGS and individual genotyping data [4]. Similarly, Kofler et al. reported that pooled sequencing accurately captures genome-wide variability patterns, showing high concordance with Sanger sequencing results [5]. By utilizing whole-genome sequencing of pooled DNA samples, this study identifies genetic variants that could support molecular-assisted selection (MAS) strategies. The adoption of Pool-seq provides an economical approach to assessing genetic diversity between these phenotypically distinct breeds [6], offering a foundation for the sustainable conservation and genetic improvement of Tong sheep.

## 2. Results

### 2.1. Whole-Genome Re-Sequencing Data

The whole-genome sequencing of Tong and Hu sheep DNA pools yielded 130.47 GB and 111.88 GB of raw data, respectively. The average sequencing depths achieved were 47.14× for the Tong sheep pool and 40.42× for the Hu sheep pool. Post-quality filtering, the datasets were refined to 126.87 GB for the Tong sheep pool and 108.76 GB for the Hu sheep pool, representing the clean data used for subsequent analyses. The GC content of the clean data was approximately 44%, which is consistent with the expected range and suggests minimal microbial contamination. Alignment of the clean reads to the Ovis aries reference genome (ARS-UI_Ramb_v3.0) showed a high alignment rate of 99.6% for both DNA pools, indicating excellent sequence correspondence with the reference genome. The sequencing data quality was confirmed with a Q20 value exceeding 97%, indicating high accuracy. Detailed metrics of the data evaluation are provided in Table 1.

### 2.2. Variant Annotation

**Figure 1:**
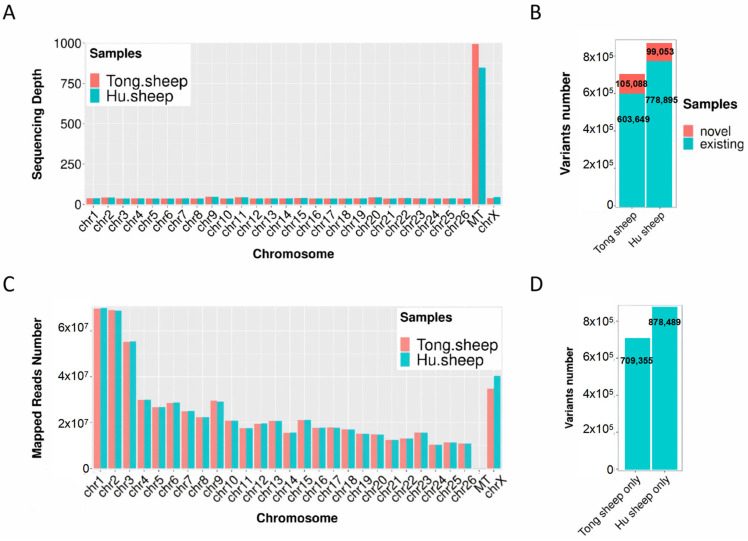
Sequencing depth, reads number, and distribution between Tong sheep and Hu sheep. (**A**) Sequencing depth of Tong sheep and Hu sheep DNA-pool; (**B**) number of novel and existing variants in Tong sheep and Hu sheep; (**C**) reads distribution of different chromosomes in Tong sheep and Hu sheep; (**D**) number of variants only in Tong sheep or Hu sheep. Notes: chr: chromosome.

In the analysis of genomic variants, a total of 708,737 variants were identified in Tong sheep, comprising 105,088 novel variants and 603,649 existing variants. Conversely, Hu sheep exhibited a total of 877,948 variants, including 99,053 novel variants and 778,895 existing variants (refer to Figure 1 for distribution). The distribution of variants within functional genomic regions was as follows: splice acceptor variants were found to be 16 in T and 19 in H, while splice donor variants numbered 15 in T and 16 in H. Variants leading to stop codon gains were observed in Tong sheep (T = 47) and Hu sheep (H = 70). Frameshift variants were also noted in both breeds (T = 3, H = 4), as were start codon losses (T = 7, H = 10). Inframe insertions and deletions were observed, with Tong sheep having eight insertions and one deletion, and Hu sheep having four insertions and four deletions. Missense variants, which result in amino acid changes, were identified in Tong sheep (T = 5185) and Hu sheep (H = 7347). Splice region variants were documented in both breeds (T = 1011, H = 1414). Other notable variant types included synonymous variants, which do not alter amino acid sequences (T = 13,804, H = 21,540), and variants in non-coding regions such as 5′ and 3′ untranslated regions (UTRs), introns, and regions upstream and downstream of genes. Specifically, variants in mature miRNA (microRNA) regions were identified, numbering five in Tong sheep and three in Hu sheep, suggesting potential impacts on gene regulation. The analysis also categorized variants based on their structural impact, including single-nucleotide variations (SNVs), insertions, deletions, and copy number variations (CNVs). SNVs were identified in Tong sheep (T = 695,383) and Hu sheep (H = 864,484). CNV analysis revealed gains (T = 10, H = 6) and losses (T = 21, H = 30) in both sheep breeds (Table 2).

### 2.3. Enrichment and Functional Regions Evaluation

**Figure 2:**
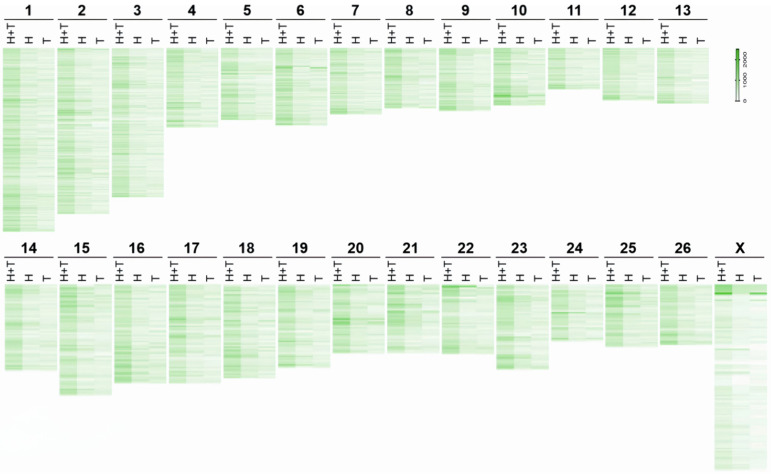
Chromosome distribution of variants only in Tong sheep and Hu sheep. Notes: (**T**) Tong sheep; (**H**) Hu sheep.

The genomic analysis revealed distinct chromosomal regions harboring variants unique to Tong and Hu sheep. A density heat-map highlighted these differences, pinpointing specific loci on chromosomes 10 (70~71 Mb and 71~72 Mb), 20 (71~72 Mb), 25 (6~7 Mb), and X (6~7 Mb), as illustrated in Figure 2. The associated genes within these regions are catalogued in Table 3. Further investigation through Gene Ontology (GO) and Kyoto Encyclopedia of Genes and Genomes (KEGG) enrichment analyses shed light on the functional implications of these variants. In Tong sheep, variants were predominantly enriched in functions related to the extracellular matrix structure, ATPase activity, and collagen type IV trimer. Notably, these variants also played roles in enhancing T cell activation, phospholipase C-activated G-protein signaling, and meiotic spindle organization. KEGG pathway analysis revealed significant involvement in ECM-receptor interactions, inositol phosphate metabolism, protein digestion and absorption, platelet activation, and the phosphatidylinositol signaling system.

**Table 3:** High-density variant regions between Tong sheep and Hu sheep.

Chromosome	Location and Variants Number	Gene Name
10	70~71 Mb (N = 1549)	LOC101106534; LOC101106781
10	71~72 Mb (N = 1662)	LOC101109370;
20	25~26 Mb (N = 1517)	GCM1; FBXO9; CILK1; LOC101107232; LOC101108696; ELOVL5; DQA; LOC101110277; LOC101110006; BTNL2; LOC101109747
22	1~2 Mb (N = 1730)	NA
25	6~7 Mb (N = 1504)	KCNK1; MAP3K21; PCNX2
X	6~7 Mb (N = 2038)	GPR143; TBL1X

**Figure 3:**
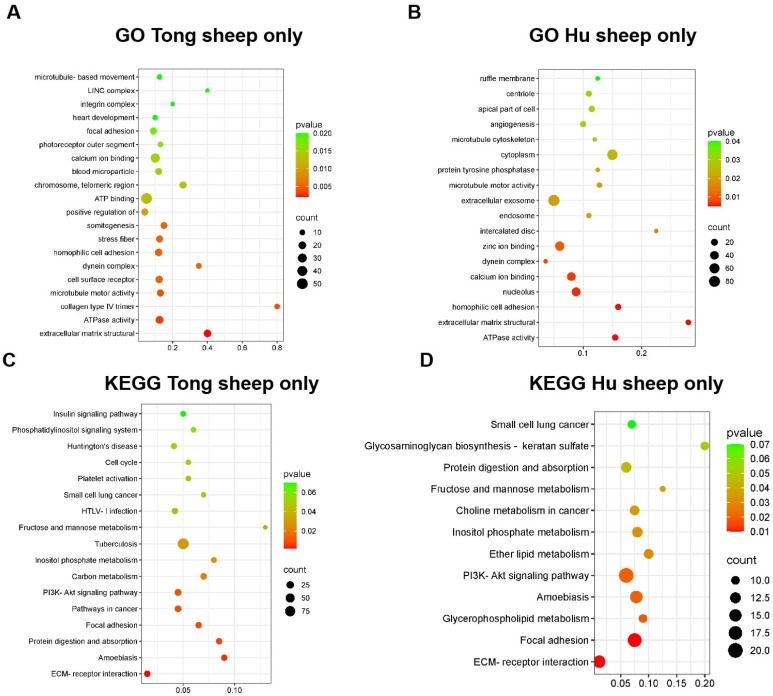
GO and KEGG enrichment of different variants within Tong sheep and Hu sheep. (**A**) GO enrichment of variants only in Tong sheep; (**B**) GO enrichment of variants only in Hu sheep; (**C**) KEGG enrichment of variants only in Tong sheep; (**D**) KEGG enrichment of variants only in Hu sheep. Notes: GO: Gene Ontology; KEGG: Kyoto Encyclopedia of Genes and Genomes.

Conversely, in Hu sheep, GO analysis identified an enrichment of variants in functions associated with the extracellular matrix, embryonic development regulation, phosphatidylinositol dephosphorylation, and ovulation. KEGG pathway analysis indicated a strong presence in ECM–receptor interaction, glycerophospholipid metabolism, ether lipid metabolism, and inositol phosphate metabolism pathways, as depicted in Figure 3.

### 2.4. miRNA and Target Genes Enrichment

**Figure 4:**
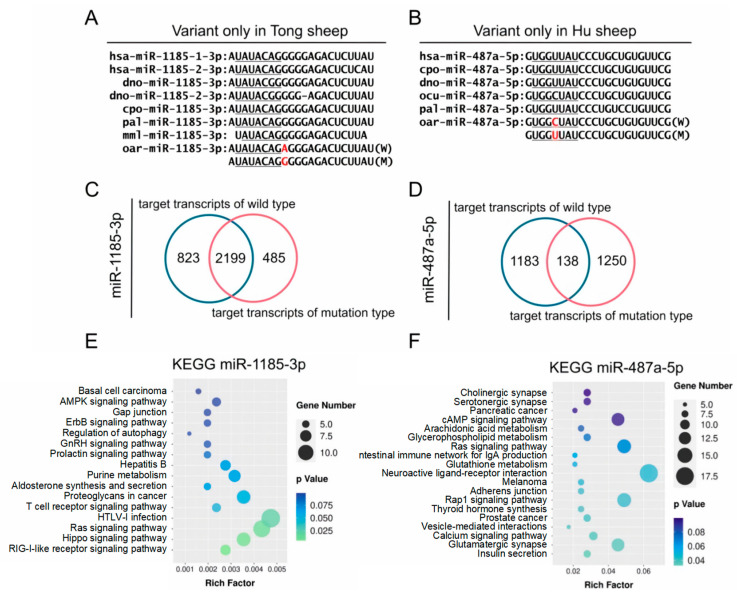
Variants located in miRNA and their functions in Tong sheep and Hu sheep. (**A**) Sequence analysis of miR-1185-3p in different species; (**B**) sequence analysis of miR-487-5p in different species; (**C**) different target transcripts caused by variants in miR-1185-3p; (**D**) different target transcripts caused by variants in miR-487-5p; (**E**) KEGG enrichment of different target transcripts caused by variants in miR-1185-3p; (**F**) KEGG enrichment of different target transcripts caused by variants in miR-487-5p. Notes: Red letters indicate mutated bases in (**A**,**B**).

**Scheme 1:**
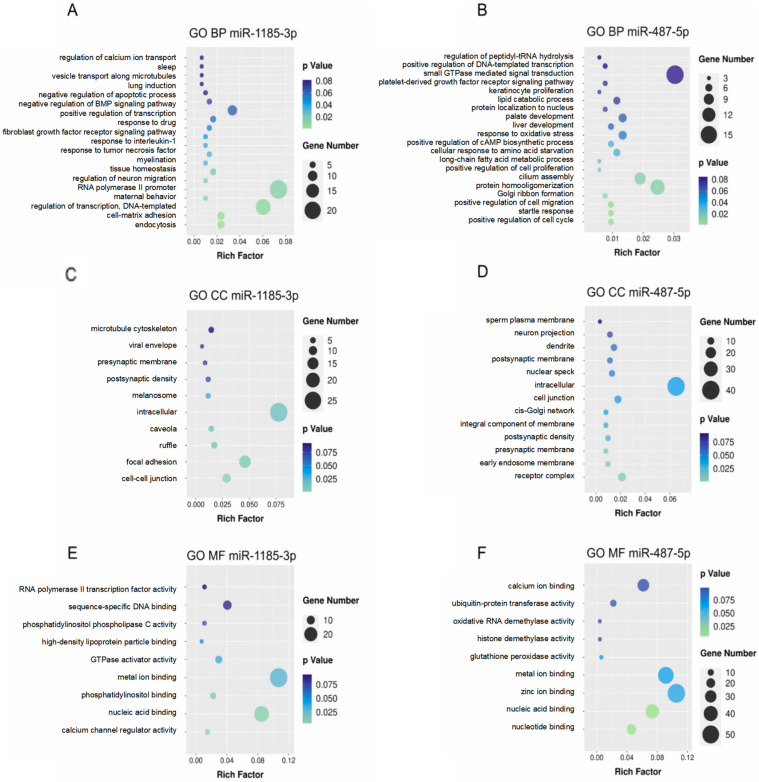
GO enrichment of target transcripts caused by variants in miR-1185-3p and miR-487-5p. (**A**) Biological process of target transcripts caused by variants in miR-1185-3p; (**B**) biological process of target transcripts caused by variants in miR-487-5p; (**C**) cellular component of target transcripts caused by variants in miR-1185-3p; (**D**) cellular component of target transcripts caused by variants in miR-487-5p; (**E**) molecular function of target transcripts caused by variants in miR-1185-3p; (**F**) molecular function of target transcripts caused by variants in miR-487-5p. Notes: BP: biological process; MF: molecular function; CC: cellular component.

In our analysis, five miRNA variants were exclusively identified in Tong sheep, and three unique variants were found in Hu sheep. A comparative review against the miRBase database (http://www.mirbase.org, accessed on 30 November 2023) highlighted three specific loci: miR-1185-3p (Tong sheep only), miR-487a-5p (Hu sheep only), and miR-655-3p (Hu sheep only), with miR-1185-3p and miR-487a-5p showing considerable conservation across mammals (Figure 4A,B). Prediction efforts revealed 1308 differential target transcripts for miR-1185-3p, 3433 for miR-487-5p, and 20 for miR-655-3p, as detailed in Figure 4C,D and Supplementary Table S1. Functional analyses through GO and KEGG pathways were conducted to elucidate the roles of these miRNA-induced transcript variations. KEGG pathway enrichment for miR-1185-3p variants predominantly involved the T cell receptor signaling and Gonadotropin-releasing hormone (GnRH) pathways (Figure 4E), while miR-487-5p variants were chiefly associated with glycerophospholipid metabolism and arachidonic acid metabolism (Figure 4F). No significant pathway enrichment was observed for miR-655-3p in KEGG analysis. The results of GO is similar to KEGG, as depicted in Scheme 1.

### 2.5. Visualization of Secondary Structure and Expression Verification

**Figure 5:**
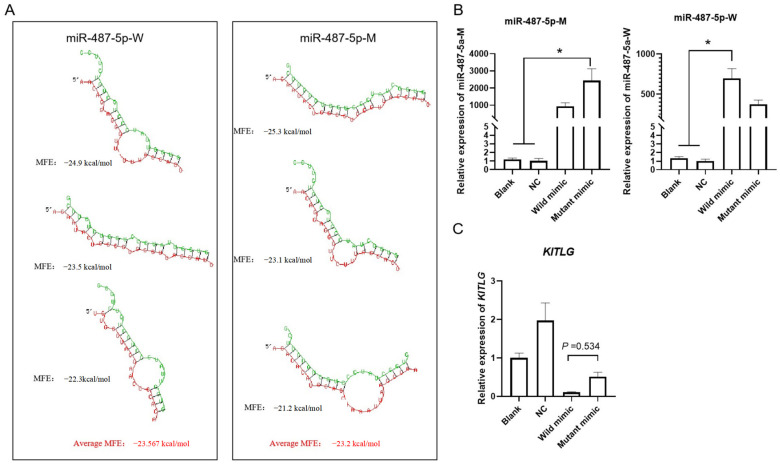
Visualization of secondary structure and expression verification. (**A**) Secondary structure and binding energy of miR-487-5p binding sites to *KITLG*; (**B**) relative expression of miR-487-5p-M and miR-487-5p-W; (**C**) relative expression of *KITLG* in wild-type mimic and mutant type mimic. * *p* < 0.05 Notes: MFE: Minimum Free Energy; Average MFE: Average Minimum Free Energy. Green letters indicate wild-type and red letters indicate mutant type.

**Scheme 2:**
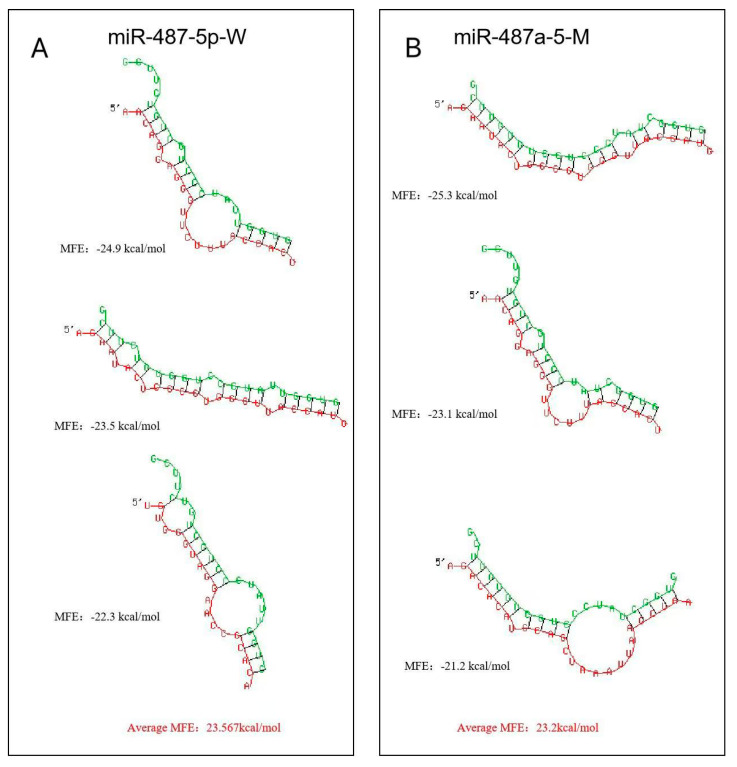
Visualization of secondary structure and expression verification. (**A**) Secondary structure and binding energy of miR-487-5p-W binding sites to *LHCGR*. (**B**) Secondary structure and binding energy of miR-487-5p-M binding sites to *LHCGR*.

According to the number of differential transcripts of miRNA and the results of functional enrichment analysis, miR-487-5p is more closely related to the regulation of phenotypic differences between breeds. We identified key genes related to lambing rate and fertility from the target genes of miR-487-5p, including *KITLG* and *LHCGR*. The binding sites and secondary structures of the 3′ UTR region of these key genes with both wild-type and mutant miRNA were predicted. Whether calculating the average binding energy across all binding sites for the target genes or considering the minimum energy value for each target gene, the binding affinity of mutant miR-487-5p to *KITLG* was significantly reduced compared to the wild-type (Figure 5A), while no significant change was observed for *LHCGR* (Scheme 2). Subsequently, by synthesizing and transfecting both miR-487-5p-W and miR-487-5p-M mimics into primary cells, the results showed that the expression of wild-type (miR-487-5p-W) and mutant-type (miR-487-5p-M) was significantly up-regulated (Figure 5B) and the expression of *KITLG* in the mutant-type group was nearly significantly up-regulated (Figure 5C).

## 3. Discussion

Despite the high fertility and growth rates characteristic of sheep, the distinct mutton odor remains a significant barrier to promoting sheep meat products. This odor arises from a combination of breeding practices, management conditions, and inherent breed traits [7]. Tong sheep, known for their minimal mutton odor under standard feeding conditions, offer promising potential for crossbreeding to enhance meat quality. However, their genetic exploitation is hindered by challenges such as low fecundity, inefficient feed conversion, and the presence of a fat tail.

Advances in genome-wide association studies (GWASs) have uncovered numerous genetic variants linked to complex traits in livestock [8,9,10,11,12]. While GWAS is a powerful tool, it has limitations in cross-population or crossbred comparisons. Sanger sequencing has identified loci of breeding value in various animal populations, including InDel loci related to growth traits in Tong sheep [13,14]. However, a deeper understanding of Tong sheep’s genetic traits requires further research. Transcriptome sequencing has offered valuable insights into breed differences [15,16,17], but the tissue- and cell-specific nature of gene expression regulation complicates the identification of genetic mechanisms underlying these distinctions [18].

We recognize that using F1 populations limits the power of BSA-seq (Bulked Segregant Analysis sequencing) to resolve causal variants and fine-map loci [19,20]. In F1 populations, heterozygosity at parental loci and the lack of additional recombination events can obscure the detection of genotype–phenotype associations. For example, the equal allele frequencies in heterozygous loci make it harder to distinguish between linked and causal variants compared to F2 populations where allele frequencies segregate in a 1:2:1 pattern [21]. To address the reduced resolution of F1-based BSA-seq, we employed a high sequencing depth (40×) to ensure reliable variant calling and minimize noise from pooled samples. Furthermore, our functional enrichment analyses of variants and miRNA targets provide additional insights into the biological pathways and candidate genes involved, which partially compensates for the limitations in mapping precision.

In this study, Hu sheep were used as a control to identify functional variants associated with fat deposition and reproductive traits through Pool-seq. While F2 populations with specific traits would provide higher resolution for BSA-seq, sample collection challenges necessitated the use of F1 populations as a second-best alternative. Despite this, Pool-seq enabled genome-wide evaluation of germplasm resources, identifying 708,737 and 877,948 variants in Tong and Hu sheep, respectively. Functional enrichment analysis highlighted 59 GO terms and 18 KEGG pathways uniquely enriched in Tong sheep, such as T cell activation and phosphatidylinositol signaling, linked to adaptive immunity and disease resistance. In Hu sheep, 45 GO terms and 13 KEGG pathways were exclusively enriched, focusing on lipid metabolism and reproduction.

Variant distribution analysis identified chromosomal regions with high variant densities, implicating genes such as *ELOVL5*, *BTNL2*, *GSTA*, and *CILK1*. *ELOVL5*, in particular, plays a crucial role in fatty acid elongation and has been associated with fat synthesis regulation [22]. Furthermore, miRNA variants affecting seed regions significantly influenced gene expression regulation [23]. Two miRNA variants were identified through Pool-seq, with functional analysis linking miR-487-5p to immune and reproductive pathways. Specifically, *KITLG* and *LHCGR,* both reproductive-related genes, were identified as targets of miR-487-5p, with mutations reducing the binding affinity of mutant miRNA to *KITLG*. This gene plays a vital role in mammalian reproduction, particularly in follicle development and ovulation [24,25,26,27,28] and is expressed at higher levels in sheep breeds with higher lambing rates [29]. Functional assays showed that mutant miRNA led to a slight but measurable increase in *KITLG* expression compared to the wild type.

The findings suggest that mutations in miR-487-5p may regulate *KITLG* expression, contributing to phenotypic differences between Tong and Hu sheep. While these results offer insights into the role of miRNA mutations in shaping breed-specific traits, they also underscore the complexity of non-coding gene regulation and its interaction with phenotypes. Future studies employing complementary methods will be crucial to validate these findings and fully harness the genetic potential of these sheep breeds.

## 4. Materials and Methods

### 4.1. Ethics Approval

All experimental protocols were conducted in strict accordance with the Regulations for the Administration of Affairs Concerning Experimental Animals, sanctioned by the State Council of the People’s Republic of China. The Institutional Animal Care and Use Committee of Northwest A&F University granted ethical approval for this study (Permit Number: NWAFAC1019).

### 4.2. Samples Collection

This research involved the collection of DNA from 50 healthy sheep, consisting of 25 female Tong sheep and 25 female Hu sheep. The Tong sheep samples were collected from the Tong sheep conservation farm located in Baishui County, Shannxi Province, China, and the Hu sheep samples were collected from a family farm in Yangling District, Shaanxi Province, China. Blood samples were drawn from the jugular vein of each animal using tubes pre-filled with sodium heparin as an anticoagulant. The blood samples were kept at −80 °C for subsequent analyses.

### 4.3. Genomic DNA Library Construction and Genome Resequencing

Genomic DNA was extracted from the leukocytes in the venous blood samples utilizing the phenol–chloroform extraction method [30]. To ensure the highest quality, DNA samples underwent rigorous quality control checks, with the optical density (OD) 260/280 ratio maintained within the ideal range of 1.8 to 2.0. Subsequently, DNA concentrations were uniformly adjusted to 100 ng/μL and preserved at −80 °C for long-term storage. Following this, equal quantities of genomic DNA from the two groups, consisting of 25 Tong sheep and 25 Hu sheep, were pooled separately as per Fracassetti et al. [31]. We conducted whole-genome sequencing on these pooled samples using the HiSeq™ Sequencer (Illumina, San Diego, CA, USA), adhering strictly to the manufacturer’s guidelines, aiming for a target coverage depth of 40× with 150 bp paired-end reads.

### 4.4. Quality Control, Variants Annotation, and Functional Enrichment

The sequencing data underwent an initial filtering process to remove adaptor sequences, reads with over 5% ambiguous bases (denoted as “N”), and low-quality reads (those with more than 20% of bases having a quality score below 20). The cleaned data were then aligned to the ARS-UI_Ramb_v3.0 sheep genome assembly using the BWA-mem algorithm with specified parameters (bwa mem -t 8 -R) [32]. Variant calling for single-nucleotide polymorphisms (SNPs) and insertion–deletions (InDels) was performed using the GATK 4.0.0 software suite (https://software.broadinstitute.org/gatk/, accessed on 20 November 2023) [33]. Copy number variations (CNVs) were estimated using CNVnator v0.4.1 [34], while structural variations were analyzed with DELLY v0.8.9 [35]. To understand the functional implications of the identified variants, we conducted an analysis based on Gene Ontology (GO)—encompassing biological processes, molecular functions, and cellular components—and the Kyoto Encyclopedia of Genes and Genomes (KEGG) pathways, utilizing the DAVID database (https://davidbioinformatics.nih.gov//, accessed on 22 November 2023).

### 4.5. Prediction of miRNA Target Genes and Enrichment Analysis

To identify the differential target transcripts influenced by miRNA variants, we employed the miRanda algorithm (miRanda v3.3a), a robust tool for miRNA target prediction. Our selection criteria for target transcripts were stringent, requiring a minimum score of 150 and a minimum free energy (MFE) threshold of less than −7 kcal/mol. The specific computational codes utilized for this analysis are detailed in Supplementary Material S1. To gain insights into the biological significance of these miRNA target genes, we conducted a comprehensive functional analysis. This included Gene Ontology (GO) annotations to classify genes into categories based on biological processes, molecular functions, and cellular components. Additionally, we explored the involvement of these genes in various biological pathways using Kyoto Encyclopedia of Genes and Genomes (KEGG) pathway analysis. Both GO annotations and KEGG pathway analyses were performed through the DAVID bioinformatics resource (https://davidbioinformatics.nih.gov/, accessed on 25 November 2023).

### 4.6. Visualization of Secondary Structure of miRNA and Target Genes

In the RNAhybrid database, we provide the sequence of miRNA and 3 ′UTR of the key target gene. The secondary structure and number of binding sites of the wild type (miR-487-5p-W) and mutant type (miR-487-5p-M) of miRNA and the genes were predicted, and the binding force was described by MFE. MFE represents the lowest free energy that these molecules need to achieve to form their most stable structure. The lower the free energy, the more stable the molecular structure or complex is.

### 4.7. Transfection of miRNA Mimics

miR-487-5p-W and miR-487-5p-M mimics were transfected into sheep mammary cell to study their regulatory effects on target gene expression. Synthetic mimics were prepared in transfection complexes using lipo8000 (Beyotime, Shanghai, China), which are then added to the cells at 60–80% confluency. qPCR analysis is conducted to measure changes in target gene expression levels after transfection for 48 h.

### 4.8. Primers Design and Real-Time Quantitative PCR (qPCR)

Total RNA was extracted using the RNAiso Plus protocol, and its quality was assessed with a Nanodrop 1000 spectrophotometer (Thermo Scientific, New York, NY, USA). cDNA synthesis was conducted in accordance with the instructions provided with the PrimeScript RT Reagent Kit (Takara, Tokyo, Japan). The primers, detailed in Table 4, were designed using the NCBI Primer-BLAST tool, referencing the Oar rambouillet v1.0 sheep genome sequence. qPCR assays were carried out using the SYBR Green PCR Master Mix (Takara, Tokyo, Japan) on a LightCycler 480 Real-Time PCR System (Roche, Basel, Switzerland). The amplification conditions were set as follows: an initial denaturation at 95 °C for 30 s, followed by 50 cycles of denaturation at 95 °C for 30 s and annealing at 60 °C for 30 s. Melting curve analysis was performed over a temperature range from 55 °C to 95 °C, with a temperature increase of 0.5 °C every 5 s. All samples were processed in triplicate. Quantification was normalized to the 18S rRNA gene, and relative gene expression levels were calculated using the 2^−ΔΔCt^ method. Statistical analysis of the qPCR data was performed using one-way ANOVA in SPSS 28.0 software (IBM SPSS Statistics, Chicago, IL, USA).

## 5. Conclusions

This study employed pooled sequencing (Pool-seq) to investigate genomic differences between Tong and Hu sheep, identifying numerous breed-specific genetic variants. These variants were enriched in key biological pathways, such as T cell activation, glycerophospholipid metabolism, and ovulation, shedding light on potential mechanisms driving differences in immunity, metabolism, and reproductive efficiency. Significantly, five chromosomal regions exhibited notable differences in variant density between the breeds, including one region harboring the *ELOVL5* gene, which plays a role in fat synthesis and deposition. Additionally, two unique miRNA variants were identified in each breed, with the mutation in miR-487-5p found to alter its regulatory interaction with the *KITLG* gene, a key player in fertility. While the study highlights potential associations through gene expression analysis and miRNA target predictions, these findings require further experimental validation to establish direct links with phenotypic traits. Future research is needed to clarify the functions and regulatory networks of these genetic elements within complex biological systems.

## Figures and Tables

**Table 1:** Overview of whole-genome re-sequencing of Tong sheep and Hu sheep.

Sample Name	Tong Sheep	Hu Sheep
Total reads	745,878,106	739,382,182
Reads filter (%)	94.66	94.52
GC (%)	44.04	43.82
Mapped	697,320,958	702,453,283
Mapped rate (%)	99.60	99.60
Unique mapped	620,684,511	627,947,209
Unique mapped rate (%)	88.70	89.10
Q20 (%)	97.09	97.04
Q30 (%)	92.63	92.51
Raw yield	130.47 G	111.88 G
Clean yield	126.87 G	108.76 G
Sequencing depth	47.14×	40.42×

**Table 2:** Consequences of variants between Tong sheep and Hu sheep.

Consequences	Tong Sheep (N)	Hu Sheep (N)
Splice acceptor variant	16	19
Splice donor variant	15	16
Stop gained	47	70
Frameshift variant	3	4
Start lost	7	10
Inframe insertion	8	4
Inframe deletion	1	1
Missense variant	5185	7347
Splice region variant	1011	1414
Stop retained variant	2	3
Synonymous variant	13,804	21,540
Coding sequence variant	5	5
Mature miRNA variant	5	3
5 prime UTR variant	327	391
3 prime UTR variant	1512	1776
Non-coding transcript exon variant	808	953
Intron variant	200,429	244,295
Non-coding transcript variant	12,601	14,798
Upstream gene variant	23,849	28,537
Downstream gene variant	21,793	25,362
Intergenic variant	441,521	548,152

Notes: N: number.

**Table 4:** Primers list for this study.

Primers Name	Sequence
miR-487-5p-W	GCTTGTGTCGTCCCTATCGGTG
	Universal PCR Primer R
miR-487-5p-M	F: GGAGGTCTGTGCTGTGAATGG
	Universal PCR Primer R
U6	F: GACCGCGCGTGTCCAGCTTA
	R: CACGAATTTGCGTGTCATCCTTGC
KITLG	F: ATGACCTTGTGGAGTGCATGGAAG
	R: GGAGTAAACTGCCTGGGTTCTGG

## Data Availability

The sequencing reads of Pool-seq were deposited in the NCBI Sequence Read Archive (https://www.ncbi.nlm.nih.gov/sra/, accessed on 30 November 2023) database with the accessions SRR13761030 and SRR13761031 under BioProject PRJNA686235.

## Supplementary Materials

The following supporting information can be downloaded at: www.mdpi.com/article/10.3390/ijms252312919/s1.

## Abbreviation

**Item**	**Definition**
MAS	Molecular-assisted selection
GWAS	Genome-wide association study
T	Tong sheep
H	Hu sheep
ELOVL5	ELOVL fatty acid elongase 5
N	Number
chr	Chromosome
GO	Gene Ontology
KEGG	Kyoto Encyclopedia of Genes and Genomes
WT	Wild type
MT	Mutation type
UTR	Untranslated regions
BP	Biological process
MF	Molecular function
CC	Cellular component
